# Microscopic Anterior Cervical Discectomy and Fusion Versus Posterior Percutaneous Endoscopic Cervical Keyhole Foraminotomy for Single-level Unilateral Cervical Radiculopathy: A Systematic Review and Meta-analysis

**DOI:** 10.1097/BSD.0000000000001327

**Published:** 2022-03-29

**Authors:** Linlin Guo, Jiajing Wang, Zhen Zhao, Jing Li, Hongyang Zhao, Yong Gao, Chao Chen

**Affiliations:** Departments of * Plastic Surgery; † Neurosurgery, Union Hospital; ‡ Department of Integrated Traditional Chinese and Western Medicine, Tongji Hospital; § Department of Orthopaedics, Union Hospital, Tongji Medical College, Huazhong University of Science and Technology, Wuhan; ∥ Department of Orthopaedics, Hefeng Central Hospital, Enshi, Hubei Province, China

**Keywords:** meta-analysis, cervical radiculopathy, anterior cervical discectomy and fusion, keyhole, microscopic, endoscopic

## Abstract

A systematic review and meta-analysis.

**Objective:**

The objective of this study was to compare the safety of microscopic anterior cervical discectomy and fusion (MI-ACDF) and posterior percutaneous endoscopic keyhole foraminotomy (PPEKF) in patients diagnosed with single-level unilateral cervical radiculopathy.

**Summary of Background Data:**

After conservative treatment, the symptoms will be relieved in about 90% of cervical radiculopathy patients. For the other one tenth of patients, surgical treatment is needed. The overall complication rate of MI-ACDF and PPEKF ranges from 0% to 25%, and the reoperation rate ranges from 0% to 20%.

**Materials and Methods:**

Electronic retrieval of studies from PubMed, Embase, and Cochrane Library was performed to identify comparative or single-arm studies on MI-ACDF and PPEKF. A total of 24 studies were included in our meta-analysis by screening according to the inclusion and exclusion criteria. After data extraction and quality assessment of the included studies, a meta-analysis was performed by using the R software. The pooled incidences of efficient rate, total complication rate, and reoperation rate were calculated.

**Results:**

A total of 24 studies with 1345 patients (MI-ACDF: 644, PPEKF: 701) were identified. There was no significantly statistical difference in pooled patient effective rate (MI-ACDF: 94.3% vs. PPEKF: 93.3%, *P*=0.625), total complication rate (MI-ACDF: 7.1% vs. PPEKF: 4.7%, *P*=0.198), and reoperation rate (MI-ACDF: 1.8% vs. PPEKF: 1.1%, *P*=0.312). However, the common complications of the 2 procedures were different. The most common complications of MI-ACDF were dysphagia and vertebral body sinking, whereas the most common complication of PPEKF was nerve root palsy.

**Conclusions:**

Both MI-ACDF and PPEKF can provide a relatively safe and reliable treatment for single-level unilateral cervical radiculopathy. The 2 techniques are not significantly different in terms of effective rate, total complication rate, and reoperation rate.

Cervical radiculopathy is characterized by cervical spine degenerative changes such as disk herniation and/or foraminal stenosis compressing the nerve roots.^1^–^4^ The typical clinical manifestations are pain in neck and one or both of the upper extremities, secondary to compression or irritation of nerve roots in the cervical spine. It can be accompanied by motor, sensory, or reflex deficits and is most common in persons 45–60 years of age, which result in terrible chronic pain and heavy economic burden for patients.^5^

Conservative treatment is usually effective for relieving symptoms for most patients. However, it does not work for some patients. For those patients, surgical operation is an excellent option. There are 2 main approaches of surgical treatment for cervical radiculopathy: anterior and posterior approaches. The most representative procedure using the anterior cervical approach is anterior cervical discectomy and fusion (ACDF), proposed firstly by Smith and Robinson in 1958.^6^ ACDF is considered by many surgeons to be the “gold standard” of surgical interventions.^7^,^8^ However, ACDF still has some catastrophic complications in spite of using the microscope, including dysphagia, adjacent segment disease, and pseudoarthrosis.^9^,^10^ The posterior cervical approach can avoid these complications caused by the anterior cervical approach. The rapid development of endoscopic technique has made the posterior cervical foraminotomy (PCF) minimally invasive, avoiding the injury of the large surgical incision to the paravertebral muscles.^11^,^12^ So posterior percutaneous endoscopic keyhole foraminotomy (PPEKF) has become an alternative technique to open discectomy in the posterior cervical approach, especially for single-level unilateral cervical radiculopathy.^13^,^14^

Few previous studies and meta-analysis have compared the therapeutic effects of ACDF and PCF in the treatment of cervical radiculopathy.^15^ Some researchers reported that the PCF method is significantly superior to the ACDF method, whereas others considered that there is no significant difference between the 2 methods in terms of treatment effect. So far, there is no meta-analysis that compared the treatment effect and safety for the single-level unilateral cervical radiculopathy by MI-ACDF and PPEKF treatment, which may be attributed to the lack of comparative studies on the 2 surgical methods. To the best of our knowledge, only 1 randomized controlled trial (RCT) has reported the therapeutic effects of MI-ACDF and PPEKF in the treatment of single-level cervical radiculopathy.^13^ Therefore, the controversy about whether MI-ACDF or PPEKF is superior for the treatment of single-level unilateral cervical radiculopathy remains to be resolved.

Given that, we conducted a meta-analysis with recent single-arm studies to compare comprehensively the effective rate, total complication rate, and reoperation rate between the 2 surgical methods.

## MATERIALS AND METHODS

### Literature Search

Electronic retrieval of articles published between January 2000 and December 2020, from PubMed, Embase, and Cochrane Library, was performed to identify comparative or single-arm studies on MI-ACDF and PPEKF. A full-text search of all studies was performed using the following Boolean search strings: (cervical radiculopathy) AND (foraminotomy OR laminoforaminotomy OR discectomy) AND (microscopic OR microendoscopic OR endoscopic OR full-endoscopic). In addition, all reference lists of the included studies were reviewed to identify potentially relevant articles.

### Eligibility Criteria

The inclusion criteria for the studies were as follows: randomized or nonrandomized controlled and case series designs, the inclusion of patients who were diagnosed with single-level unilateral cervical radiculopathy, analysis of patients conservatively treated for >4 weeks, surgical intervention with MI-ACDF or PPEKF, and detailed reporting of outcomes of complications and/or reoperation.

The exclusion criteria were as follows: enrollment of <10 patients; inclusion of patients with multilevel or bilateral cervical radiculopathy; inclusion of patients whose diagnosis encompassed cervical myelopathy, tumor, tuberculosis, fraction, infection, or malformation; inclusion of patients with a history of previous cervical surgery; keyhole foraminotomy using the anterior approach; cadaveric or biomechanical studies; and case reports, reviews, editorials, letters, or commentary articles.

### Data Extraction

The following data were extracted from the selected studies: (1) first author, year of publication, and study region; (2) demographic features of participants, including sample size, male/female ratio, and mean age; (3) surgical methods; (4) duration of follow-up; (5) the number of people who were completely cured and obviously remission; (6) rate of overall complications and specific categories; and (7) number of reoperations and their reasons.

### Quality Assessment

The Newcastle-Ottawa Scale (NOS) was used to assess the quality of nonrandomized comparative studies. NOS includes the evaluation criteria of nonrandomized comparative studies, which are mainly divided into 3 categories: the selection of study groups, comparability between study groups, and outcome evaluation. The system uses a “star system” with 8 entries and has a full score of 9 stars. RCTs and nonrandomized studies with 6 or more stars were considered to be of studies with relatively high quality. Studies evaluated as low quality were excluded from this meta-analysis.

Two researchers independently performed the literature search, inclusion of qualified studies, quality evaluation, and data extraction (Z.Z., J.W.). When disagreements occurred, a consensus was reached through consultation with the third senior professor (C.C.).

### Statistical Analysis

Two independent investigators (L.G., J.L.) performed the statistical analysis using the data-processing software program R GUI 3.6.1. The incidence rate and 95% confidence interval (CI) were calculated using a 0.5 cell correction for studies with zero event endpoints. And since the incidence rate of the original data was not in the range of 30% to 70%, it was first converted by the Arcsine transformation method to make it conform to the normal distribution. The *t* test and Higgins *I* ^2^ statistics were used to assess heterogeneity, defined as the percentage of the error caused by differences between studies. We classified heterogeneity as follows: *I* ^2^ <25%, no heterogeneity; *I* ^2^=25%–50%, low heterogeneity; *I* ^2^=50%–75%, moderate heterogeneity; and *I* ^2^ >75%, high heterogeneity. *I* ^2^ <50% generally indicates roughly consistent results and uniform research using the fixed-effects model. *I* ^2^ >50% was used as the threshold to show significant heterogeneity, and the random-effects model was selected. Subgroup analysis was tried to find the source of heterogeneity. Sensitivity analysis was performed by eliminating single studies and determining whether the other pooled results changed significantly. Funnel plot and Egger linear regression asymmetry tests were used to evaluate publication bias (*P*<0.05 was considered significant).

## RESULTS

### Search Results

According to the search sentence, a total of 813 studies were retrieved in 3 databases. After the duplicate investigation, 209 duplicate studies were excluded. Of the remaining 604 studies, 559 were excluded after the initial title, and abstract screening for that are conference abstracts, review articles, letters, laboratory studies, cadaver studies, and studies with irrelevant exposure or results.

**FIGURE 1:**
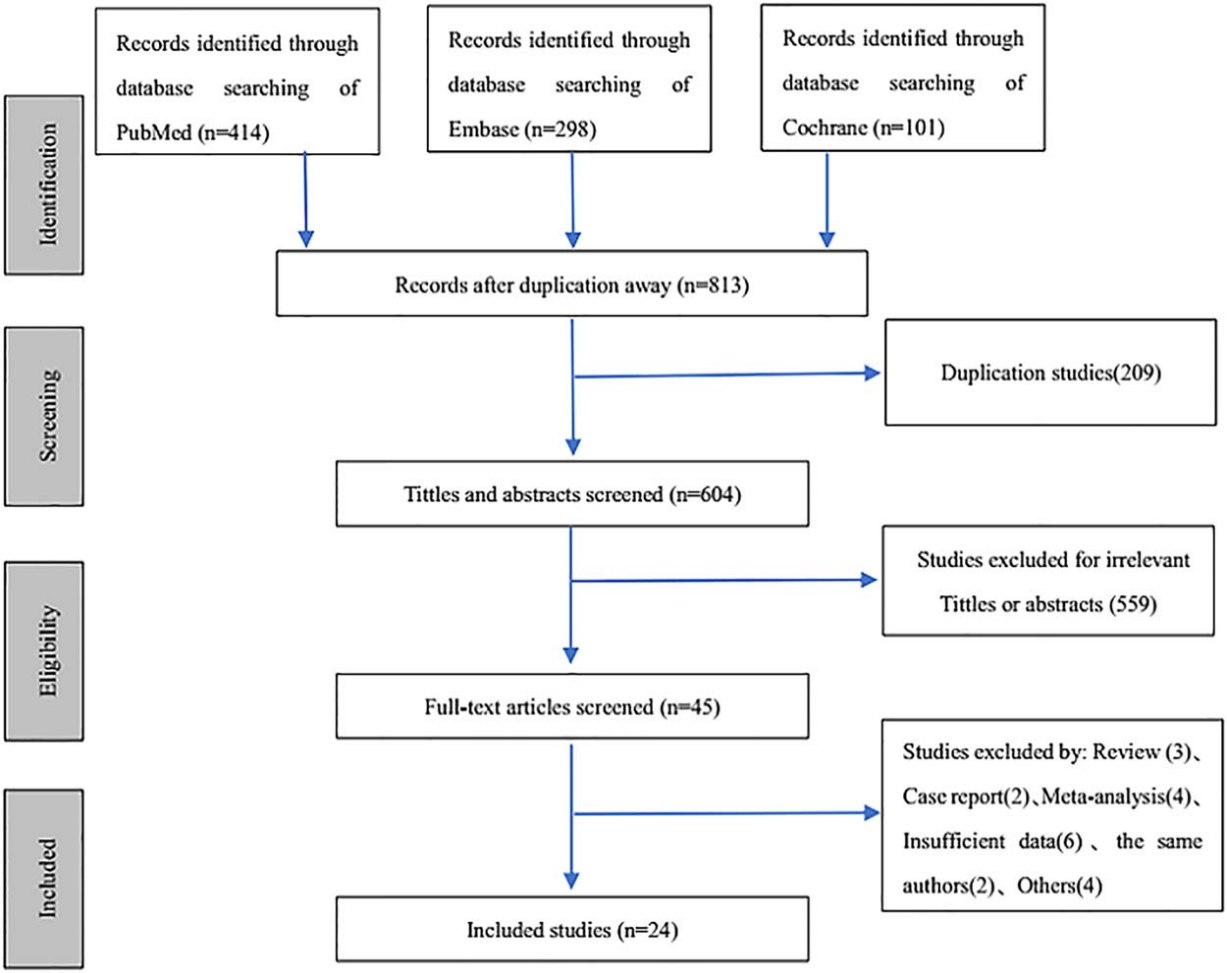
Flowchart of literature search and selection.

The full texts of the remaining 45 articles were evaluated in detail, and 21 articles were excluded according to the eligibility criteria. Furthermore, the studies reported by the same authors may have common patients, so we excluded them. Finally, our meta-analysis included 24 articles. Among the included articles, 1 randomized controlled study simultaneously reported the results of the 2 methods, and 1 randomized controlled study reported the results of only 1 of the 2 surgical methods of interest. The results were separately analyzed in this meta-analysis. The flow diagram of the study inclusion process is shown in Figure 1.

### Characteristics of the Included Studies

From the 24 studies finally identified, the meta-analysis included 1345 participants (MI-ACDF: 644, PPEKF: 701). Ruetten et al^13^ reported a direct comparison of these 2 surgical methods in a randomized controlled study; Lin et al^16^ retrospectively evaluated the clinical and radiologic results of ACDF, cervical disk replacement, and PCF; and the other studies reported 1 of the 2 surgical methods (either MI-ACDF or PPEKF), resulting in 10 studies on MI-ACDF and 16 studies on PPEKF. For one randomized controlled study, we included only the results of MI-ACDF in our study: Wirth et al^17^ reported the therapeutic effects of PCF and anterior cervical discectomy with and without fusion (all operations were performed under the microscope). The study population of MI-ACDF was 55.6% men, with an average age of 45.53 years and an average follow-up time of 32.8 months. Comparatively, the study population of MI-ACDF was 56.6% men, with an average age of 50.00 years and an average follow-up time of 16.0 months. The basic characteristics of the included studies are shown in Table 1.

**TABLE 1:** Baseline Characteristics of the Included Studies

References	Study Type	Country	Surgical Method	Sample Size	Male/Female	Mean Age	Follow-up
Ruetten et al^13^	RCT	Germany	MI-ACDF	86	NA	NA	24
Ahn et al^18^	Cohort	Korea	MI-ACDF	65	36/28	47.5	60
Scholz et al^19^	Retrospective	Germany	MI-ACDF	40	20/20	50	33
Lin et al^16^	Retrospective	Korea	MI-ACDF	55	31/24	52.5	12
Türeyen^20^	Retrospective	Turkey	MI-ACDF	43	24/19	43	18
Chen et al^21^	Prospective	China	MI-ACDF	92	50/42	46.7	24
Omidi-Kashani et al^22^	Retrospective	Iran	MI-ACDF	68	20/48	41.2	31.25
Nemoto et al^23^	RCT	Japan	MI-ACDF	46	42/4	41.23	24
Korinth et al^24^	Retrospective	Germany	MI-ACDF	124	73/51	45.9	NA
Wirth et al^17^	RCT	United States	MI-ACDF	25	14/11	41.7	69
Ruetten et al^13^	RCT	Germany	PPEKF	89	NA	NA	24
Xiao et al^25^	Retrospective	China	PPEKF	84	38/46	54.15	12
Liao et al^26^	Prospective	Germany	PPEKF	44	28/16	48.95	12
Shu et al^27^	Retrospective	China	PPEKF	32	14/18	63.0	12
Park et al^28^	Retrospective	Korea	PPEKF	13	5/8	47.1	14.8
Yao et al^12^	Retrospective	China	PPEKF	24	10/14	45.6	15.6
Won et al^11^	Retrospective	Korea	PPEKF	46	30/16	49.3	25.8
Kim et al^29^	Retrospective	Korea	PPEKF	32	22/10	49	12
Yang et al^30^	Cohort	China	PPEKF	42	28/14	40.5	12
Lin et al^16^	Retrospective	Korea	PPEKF	21	14/7	53.4	12
Ji-Jun et al^31^	Cohort	China	PPEKF	43	28/15	46.6	18
Wan et al^32^	Retrospective	China	PPEKF	25	14/11	38	12
Gushcha et al^33^	Retrospective	Russia	PPEKF	25	NA	NA	NA
Zhang et al^34^	Retrospective	China	PPEKF	42	28/14	47.1	15
Haijun et al^35^	Retrospective	China	PPEKF	106	56/50	61.21	NA
Luo et al^36^	Retrospective	China	PPEKF	33	17/16	56	25.7

MI-ACDF indicates anterior microscope cervical discectomy and fusion; NA, not available; PPEKF, posterior percutaneous full-endoscopic approach keyhole foraminotomy; RCT, randomized controlled trial.

### Quality Assessment Results

The meta-analysis included 2 randomized controlled studies and 22 nonrandomized controlled studies (most of which were retrospective studies). After the NOS quality evaluation, all nonrandomized controlled studies received >6 stars, indicating that the quality of the studies was very high, which also laid the foundation for a high-quality meta-analysis. The detailed quality evaluation results for the included studies are shown in Table 2.

**TABLE 2:** Quality Assessment of Nonrandomized Comparative Studies

References	Selection	Comparability	Outcome	Total
Ahn et al^18^	4	1	3	8
Scholz et al^19^	3	1	3	7
Lin et al^16^	4	1	3	8
Türeyen^20^	3	1	3	7
Chen et al^21^	3	1	2	6
Omidi-Kashani et al^22^	3	1	2	6
Nemoto et al^23^	4	1	3	8
Korinth et al^24^	4	1	3	8
Xiao et al^25^	3	1	3	7
Liao et al^26^	3	1	3	7
Shu et al^27^	3	1	3	7
Park et al^28^	3	1	3	7
Yao et al^12^	4	1	3	8
Won et al^11^	3	1	3	7
Kim et al^29^	4	1	3	8
Yang et al^30^	3	1	3	7
Ji-Jun et al^31^	3	1	3	7
Wan et al^32^	3	1	3	7
Gushcha et al^33^	4	1	3	8
Zhang et al^34^	3	1	3	7
Haijun et al^35^	4	1	2	7
Luo et al^36^	4	1	2	7

### Meta-analysis Outcomes

#### Effective Rate

**FIGURE 2:**
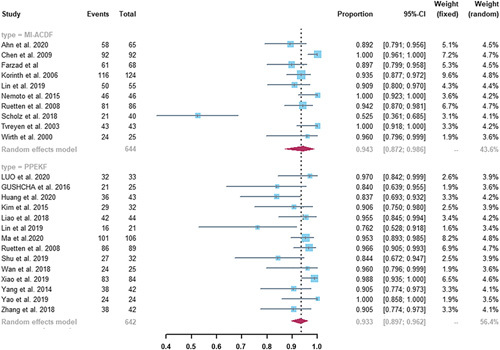
Forest plots of pooled effective rate. There was no statistical difference between MI-ACDF and PPEKF (94.3% vs. 93.3%, *P*=0.625). CI indicates confidence interval; MI-ACDF, microscopic anterior cervical discectomy and fusion; PPEKF, posterior percutaneous endoscopic keyhole foraminotomy.

Taking into account the different descriptions of these studies, thus the definition of effective rate was unified as the number of people who were completely cured and obviously remission account for the total number of patients 2 weeks after the operation. There were 10 studies of MI-ACDF group and 14 studies of PPEKF group in the all studies reported effective rate. The results showed that the effective rate was 94.3% (95% CI, 87.2%–98.6%) for the MI-ACDF group and 93.3% (95% CI, 89.7%–96.2%) for the PPEKF group, which was observed no statistically significant difference between the 2 groups (Fig. 2, *P*=0.625). A high heterogeneity between studies was noted in the MI-ACDF group (*I* ^2^=90%), and a moderate degree of heterogeneity was observed in the PPEKF group (*I* ^2^=61%).

#### Total Complication Rate

**FIGURE 3:**
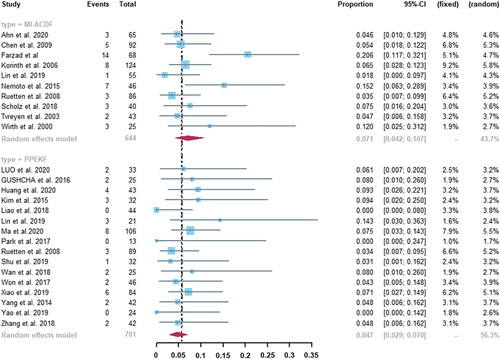
Forest plots of pooled total complication rate. There was no statistical difference between MI-ACDF and PPEKF (7.1% vs. 4.7%, *P*=0.198). CI indicates confidence interval; MI-ACDF, microscopic anterior cervical discectomy and fusion; PPEKF, posterior percutaneous endoscopic keyhole foraminotomy.

As shown in Figure 3, complications occurred in 49 of the 644 patients who underwent MI-ACDF and in 40 of the 701 patients who underwent PPEKF. The incidence of total complications in the MI-ACDF group was 7.1% (95% CI, 4.2%–10.7%), and that in the PPEKF group was 4.7% (95% CI, 2.9%–7.0%). No statistically significant difference was observed between the 2 groups (*P*=0.198). A moderate degree of heterogeneity between studies was observed in the MI-ACDF group (*I* ^2^=61%); however, a low degree of heterogeneity was noted in the PPEKF group (*I* ^2^=39%).

#### Reoperation Rate

**FIGURE 4:**
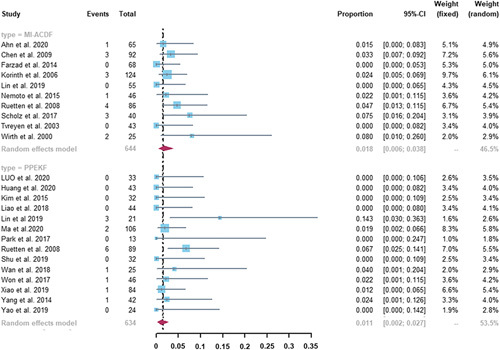
Forest plots of pooled reoperation rate. There was no statistical difference between MI-ACDF and PPEKF (1.8% vs. 1.1%, *P*=0.312). CI indicates confidence interval; MI-ACDF, microscopic anterior cervical discectomy and fusion; PPEKF, posterior percutaneous endoscopic keyhole foraminotomy.

As shown in Figure 4, a total of 24 studies provided data on reoperation, 10 of which reported on reoperation after MI-ACDF and 14 reported on reoperation after PPEKF. The incidence of total complications was 1.8% (95% CI, 0.6%–3.8%) in the MI-ACDF group and 1.1% (95% CI, 0.2%–2.7%) in the PPEKF group. No statistically significant difference was found between the 2 groups (*P*=0.312). The moderate heterogeneity was both observed in the MI-ACDF group (*I* ^2^=57%) and the PPEKF group (*I* ^2^=52%).

#### Subgroup Analysis

The results of subgroup analyses of effective rate, reoperation rate, and total complication rate according to age, duration of follow-up, and geographic region are presented as follows. In terms of efficient rate, age, follow-up time, and geographic region are not the source of heterogeneity of the MI-ACDF group, but follow-up time and geographic region may be the source of heterogeneity of the PPEKF group (Table 3). However, in terms of total complication rate and reoperation rate, age, follow-up time, and geographic region are both the source of heterogeneity of MI-ACDF and PPEKF (Tables 4, 5).

**TABLE 3:** The Subgroup Analysis of Effective Rate for MI-ACDF and PPEKF

Subgroup	Events	Total	R (95% CI)	*I* ^2^ (%)	*P*
MI-ACDF					
All studies	592	644	90% (87.2%–98.6%)	90	<0.01
Age (y)					
<50	521	549	97% (92.6%–99.5%)	82	<0.01
≥50	71	95	74.3% (31.4%–99.3%)	95	<0.01
No report	0	0	—	—	—
Follow-up time (mo)					
<24	209	222	96.3% (87.2%–98.6%)	81	<0.01
≥24	383	422	93.1% (81.7%–99.2%)	92	<0.01
Geographic region					
Asia	350	369	97.4% (90.9%–100%)	87	<0.01
Non-Asia	242	275	87.3% (69.6%–98%)	92	<0.01
PPEKF					
All studies	592	642	93.3% (89.7%–96.2%)	61	<0.01
Age (y)					
<50	231	252	93.2% (88%–97%)	52	0.05
≥50	259	276	93.2% (85.5%–98.1%)	75	<0.01
No report	107	114	92.2% (76.8%–99.6%)	75	0.05
Follow-up time (mo)					
<24	479	520	92.4% (88%–95.9%)	65	<0.01
≥24	118	122	96.7% (92.8%–99.1%)	0	0.92
Geographic region					
Asia	448	484	93.1% (88.5%–96.6%)	68	<0.01
Non-Asia	149	158	94% (87.1%–98.3%)	51	0.13

CI indicates confidence interval; MI-ACDF, microscopic anterior cervical discectomy and fusion; PPEKF, posterior percutaneous endoscopic keyhole foraminotomy; R, rate.

**TABLE 4:** The Subgroup Analysis of Total Complication Rate for MI-ACDF and PPEKF

Subgroup	Events	Total	R (95% CI)	*I* ^2^ (%)	*P*
MI-ACDF					
All studies	49	644	7.1% (4.2%–10.7%)	61	<0.01
Age (y)					
<50	45	549	8% (4.6%–12.2%)	63	<0.01
≥50	4	95	4% (0.4%–11.1%)	47	0.17
No report	0	0	—	—	—
Follow-up time (mo)					
<24	11	222	4.6% (2.1%–8.1%)	11	0.32
≥24	38	422	8.8% (4.6%–14.1%)	66	<0.01
Geographic region					
Asia	32	369	7.6% (3.1%–13.9%)	75	<0.01
Non-Asia	17	275	0.6% (3.5%–9.1%)	0	0.47
PPEKF					
All studies	40	701	4.9% (3.4%–6.6%)	39	0.06
Age (y)					
<50	15	311	3.3% (1%–6.9%)	54	0.03
≥50	20	276	7.1% (4.3%–10.4%)	0	0.67
No report	5	114	4.2% (1.3%–8.7%)	0	0.37
Follow-up time (mo)					
<24	33	533	5.2% (3.4%–7.2%)	49	0.02
≥24	7	168	4.1% (1.6%–7.6%)	0	0.82
Geographic region					
Asia	35	543	5.8% (4%–8%)	13	0.32
Non-Asia	5	158	2.2% (0%–8.7%)	68	0.08

CI indicates confidence interval; MI-ACDF, microscopic anterior cervical discectomy and fusion; PPEKF, posterior percutaneous endoscopic keyhole foraminotomy; R, rate.

**TABLE 5:** The Subgroup Analysis of Reoperation Rate for MI-ACDF and PPEKF

Subgroup	Events	Total	R (95% CI)	*I* ^2^ (%)	*P*
MI-ACDF					
All studies	17	644	1.8% (0.6%–3.8%)	57	0.01
Age (y)					
<50	14	549	1.9% (0.6%–3.8%)	49	0.06
≥50	3	95	1.8% (0%–15.7%)	86	<0.01
No report	0	0	—	—	—
Follow-up time (mo)					
<24	3	222	0.4% (0%–3.1%)	63	0.07
≥24	14	422	2.8% (0.9%–5.5%)	52	0.05
Geographic region					
Asia	5	369	0.6% (0%–2.3%)	47	0.1
Non-Asia	12	275	4.1% (0.6%–3.8%)	0	0.43
PPEKF					
All studies	15	634	1.1% (0.2%–2.7%)	52	0.01
Age (y)					
<50	3	269	0.5% (0%–1.6%)	2	0.42
≥50	6	276	1.4% (0%–4.6%)	60	0.04
No report	6	89	6.7% (2.5%–12.8%)	—	—
Follow-up time (mo)					
<24	8	466	0.9% (0.2%–1.9%)	40	0.08
≥24	7	168	2.2% (0%–8.6%)	71	0.03
Geographic region					
Asia	9	501	1% (0.3%–2.1%)	34	0.12
Non-Asia	6	133	1.9% (0%–14.7%)	88	<0.01

CI indicates confidence interval; MI-ACDF, microscopic anterior cervical discectomy and fusion; PPEKF, posterior percutaneous endoscopic keyhole foraminotomy; R, rate.

#### Common Complications of MI-ACDF and PPEKF

According to the literature reading and data analysis, the most common complications of MI-ACDF mainly included transient dysphagia, vertebral body sinking, and wound infection (Table 6). A total of 8 patients from 4 studies were complained of transient dysphagia after surgery. Among them, 3 patients had transient dysphagia recovered 2 days, 1 patient recovered 2 weeks later, and 3 patients recovered after 1 month. The remaining 1 patient also had transient dysphagia but without reporting the recovered time. There were 7 patients detected vertebral body sinking in the MI-ACDF group, including 1 patients underwent reoperation, 6 patients of mild vertebral body sinking without revision surgery. Only 1 patient underwent a second operation due to infection, and the other infected patients were healed by using the escalated antibiotics.

**TABLE 6:** Complication Rates of Studies Included in the Microscopic Anterior Cervical Discectomy and Fusion

	Overall Complications		Reoperation		Transient Dysphagia		Vertebral Body Sinking		Others	
References	n	Incidence (%)	n	Incidence (%)	n	Incidence (%)	n	Incidence (%)	n	Incidence (%)
Ruetten et al^13^	3	4.7	4	4.7	3	3	—	—	—	—
Ahn et al^18^	3	4.7	1	1.6	3	4.7	—	—	—	—
Scholz et al^19^	3	7.5	3	7.5	—	—	—	—	—	—
Lin et al^16^	1	1.8	0	0	—	—	—	—	1	1.8
Türeyen^20^	2	4.7	0	0	1	2.3	—	—	1	2.3
Chen et al^21^	5	5.4	3	3.3	—	—	—	—	5	5.4
Omidi-Kashani et al^22^	14	20.6	1	1.5	1	1.5	7	10.3	10	16.2
Nemoto et al^23^	7	15.2	0	0	—	—	—	—	—	—
Korinth et al^24^	8	6.5	3	2.4	—	—	—	—	—	—
Wirth et al^17^	3	12	2	8	—	—	—	—	3	12

— indicates no report.

In contrast, transient root injury, dural injury, and wound infection were the main complications of PPEKF (Table 7). There were 24 patients experienced transient root injury in 10 included studies. Among them, 10 patients were not reported the prognostic results, 9 patients recovered at different time after conservative treatment, 2 patients were complained intermittent pain in long term without need of special interventions, the others underwent reoperation after failure of medication treatment. In terms of dural injury, 5 patients were found dural injury in 3 included studies. Two patients were cured without surgical intervention, and the other 3 underwent secondary dural repair surgery. All infected patients recovered after antibiotic treatment.

**TABLE 7:** Complication Rates of Studies Included in the Posterior Percutaneous Endoscopic Keyhole Foraminotomy

	Overall Complications		Reoperation		Transient Root Palsy		Dural Tear		Others	
References	n	Incidence (%)	n	Incidence (%)	n	Incidence (%)	n	Incidence (%)	n	Incidence (%)
Ruetten et al^13^	3	3	6	6.7	3	3	—	—	—	—
Xiao et al^25^	6	7.1	1	1.2	6	7.1	—	—	—	—
Liao et al^26^	0	0	0	0	0	0	—	—	—	—
Shu et al^27^	1	3.1	0	0	1	3.1	—	—	—	—
Park et al^28^	0	0	0	0	0	0	0	0	—	—
Yao et al^12^	0	0	0	0	0	0	0	0	—	—
Won et al^11^	2	4.3	1	2.2	0	0	0	0	2	4.3
Kim et al^29^	3	9.4	0	0	2	6.3	1	3.1	—	—
Yang et al^30^	2	4.8	1	2.4	1	2.4	—	—	—	—
Lin et al^16^	3	14.3	3	14.3	—	—	—	—	—	—
Ji-Jun et al^31^	4	9.3	0	0	2	4.7	2	4.7	—	—
Wan et al^32^	2	8	1	4	—	—	—	—	1	4
Gushcha et al^33^	2	8	—	—	2	8	—	—	—	—
Zhang et al^34^	2	4.8	—	—	2	2.4	—	—	—	—
Haijun et al^35^	8	7.5	2	1.9	4	3.8	0	0	2	1.9
Luo et al^36^	2	6.1	0	0	1	3	2	6.1	—	—

— indicates no report.

#### Sensitivity Analysis and Publication Bias

**FIGURE 5:**
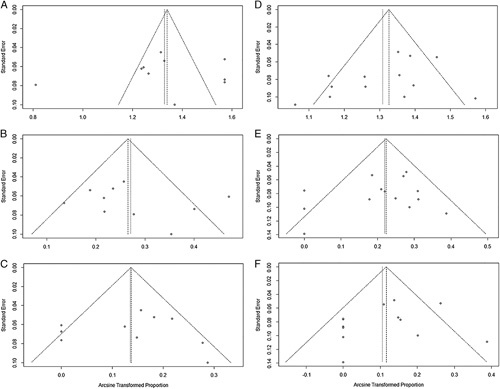
Funnel plots of SE by logit event rate. Each circle represents an identified study. A, The funnel plot of effective rate in MI-ACDF group. B, The funnel plot of total complication rate in MI-ACDF group. C, The funnel plot of reoperation rate in MI-ACDF group. D, The funnel plot of effective rate in PPEKF group. E, The funnel plot of total complication rate in PPEKF group. F, The funnel plot of reoperation rate in PPEKF group. The plots are all basically symmetric about the mean effect, which indicates an absence of substantial publication bias. MI-ACDF indicates microscopic anterior cervical discectomy and fusion; PPEKF, posterior percutaneous endoscopic keyhole foraminotomy.

There was no significant impact on the overall results by the individual elimination of each study. The Figure 5 showed that all funnel plots were not obvious asymmetry. In addition, the Egger test results again showed no substantial publication bias (all *P*>0.05). Therefore, the sensitivity analysis and publication bias tests confirmed that the results of the meta-analysis are reliable.

## DISCUSSION

Conservative treatment is provided first for cervical radiculopathy. However, if the symptoms do not considerably improve after conservative treatment for >4 weeks, surgical treatment should be considered. Nowadays, 2 major traditional surgical methods are available for single-level cervical radiculopathy: ACDF and PCF. ACDF has been widely popular with spinal surgeons due to its wide application range, high relief rate, and short learning curve.^37^–^39^ However, there are also some complications accompanied such as dysphagia, vertebral body sinking, and wound infection. MI-ACDF is a modified procedure performed under a microscope, which reduces some complications caused by dissection and separation of soft tissues. In contrast, open PCF can cause significant damage to the posterior muscles of the neck, leading to axial pain after surgery. To avoid serious complications caused by open surgery, Ruetten et al^40^ first investigated the treatment effect of posterior percutaneous full-endoscopic keyhole cervical foraminotomy, and found that PPEKF achieved the same clinical treatment effect as open surgery while reducing the complications associated with open surgery.^41^,^42^ Since it may be confusing for surgeons to choose MI-ACDF or PPEKF for single-level unilateral cervical radiculopathy, we comprehensively reviewed the recent studies on these 2 techniques in terms of effective rate, total complication rate, and reoperation rate. This systematic review and meta-analysis included a total of 24 studies involving 1345 patients.

The clinical effective rate of MI-ACDF was 94.3%, which agrees with those reports that the effective rate ranged from 80% to 100%.^9^,^43^,^44^ Similarly, the effective rate of PPEKF was also consistent with previous studies.^45^,^46^ The pooled effective rate of patients after MI-ACDF was higher than patients after PPEKF, but no statistical difference was observed between the 2 groups. The result was similar with the report by Ruetten et al^13^ that treatment effect with ACDF is same as PPEKF. Though, Lin et al^16^ reported that the therapeutic effect of ACDF is significantly better than that of PPEKF.

Our meta-analysis found that the overall complication incidence for single-level unilateral cervical radiculopathy was 5.6% in MI-ACDF and 4.8% in PPEKF, without statistical difference (*P*=0.198). Chen et al^21^ conducted a prospective study involving 92 patients and found that the overall complication rate after MI-ACDF was 5.4%. Korinth et al^24^ also reported that the overall complication rate of MI-ACDF was 6.5%. We found that studies with large sample sizes reported a complication rate that was consistent with the overall complication rate of MI-ACDF obtained in this meta-analysis. Owing to the large difference in sample sizes of the studies on PPEKF, the overall postoperative complications greatly varied. Some studies did not report any complications,^12^,^26^,^28^ but one study reported an overall complication rate as high as 14.3%.^16^ However, the specific proportions of several common complications showed significant differences. According to a comparative analysis, the most common complication in MI-ACDF was vertebral body sinking, followed by dysphagia. In PPEKF, transient root palsy and dural injury were the most common complications, as proved in the previous meta-analysis by Wu et al.^47^

No statistical difference in reoperation rate was found between MI-ACDF and PPEKF (*P*=0.312). With respect to MI-ACDF treatment, there are many reasons for recurrence or reoperation, including adjacent segment disease, graft failure, incomplete removal of the nucleus pulposus, foraminal stenosis, and incorrect postoperative rehabilitation training. In contrast, the common risk factors for reoperation in PPEKF treatment include incomplete removal of the nucleus pulposus, destruction of the intraoperative annulus, and cerebrospinal fluid leakage. Many studies have shown that the most common reason is the incomplete removal of the herniated intervertebral disk tissue during the operation and excess or deficient residual nucleus pulposus. Studies have also demonstrated that the causes of reoperation for the endoscopic transforaminal lumbar intervertebral disk are the same.^48^,^49^

This meta-analysis results showed high reliability, and we anticipated that the findings of our study can provide clarity to clinicians with respect to choosing appropriate surgical treatment for patients with single-level unilateral cervical radiculopathy. But, this meta-analysis also had some limitations. First, not all of the included studies were RCT comparative studies of MI-ACDF and PPEKF. Most of the included studies reported only 1 of the 2 surgical methods, which might have caused a certain deviation in the research results. Second, this meta-analysis only studied the effective rate, total complication, and reoperation rate of the 2 surgical methods but did not report variations in Visual Analog Scale scores, Neck Disability Index, and radiologic results. Third, this study did not make a unified definition for overall postoperative complications, which might have led to a selection bias. Last, the results might also have been affected by the surgical experience of the surgeons.

## CONCLUSIONS

In this meta-analysis, the findings showed no difference in treatment outcomes between MI-ACDF and PPEKF for patients with single-level cervical radiculopathy by evaluating the effectiveness, total complication rate, and reoperation rate. Thus, both MI-ACDF and PPEKF can provide a relatively safe and reliable treatment for single-level unilateral cervical radiculopathy.
